# Supplementary material for: Vibrationally excited molecular hydrogen production from the water photochemistry
Source: Nat Commun. 2021 Nov 2;12:6303. doi: 10.1038/s41467-021-26599-9 (PMC8563719; doi:10.1038/s41467-021-26599-9)
Supplement: Supplementary file 1 — Supplementary Information [file 41467_2021_26599_MOESM1_ESM.pdf]

Supplementary Information

Vibrationally Excited Molecular Hydrogen Production from the Water Photochemistry

Chang et al

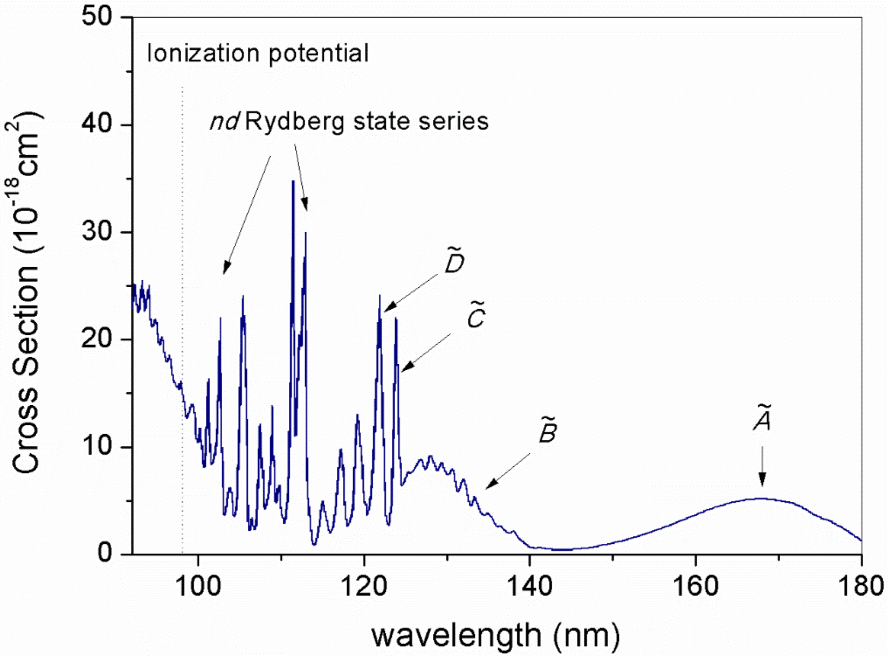

Supplementary Figure 1. Absorption spectrum of H_2O vapor at room temperature. The absorption spectrum in the ~190-120 nm region consists of two broad, well-separated bands with maxima at ~167 nm and ~128 nm, respectively. These bands are assigned to, respectively, the $\tilde{A}^1B_1 \leftarrow \tilde{X}^1A_1$ ($3sa_1 \leftarrow 1b_1$) and $\tilde{B}^1A_1 \leftarrow \tilde{X}^1A_1$ ($3sa_1 \leftarrow 3a_1$) transitions. The first intense, sharp bands peaking at ~124 nm and ~122 nm are attributed to, respectively, the $\tilde{C}^1B_1 \leftarrow \tilde{X}^1A_1$ ($3pa_1 \leftarrow 1b_1$) and $\tilde{D}^1A_1 \leftarrow \tilde{X}^1A_1$ ($3pb_1 \leftarrow 1b_1$) Rydberg transitions and most of the sharp features in the ~118-92 nm region are attributed to $nd \leftarrow 1b_1$ ($n \geq 3$) excitations.[1]

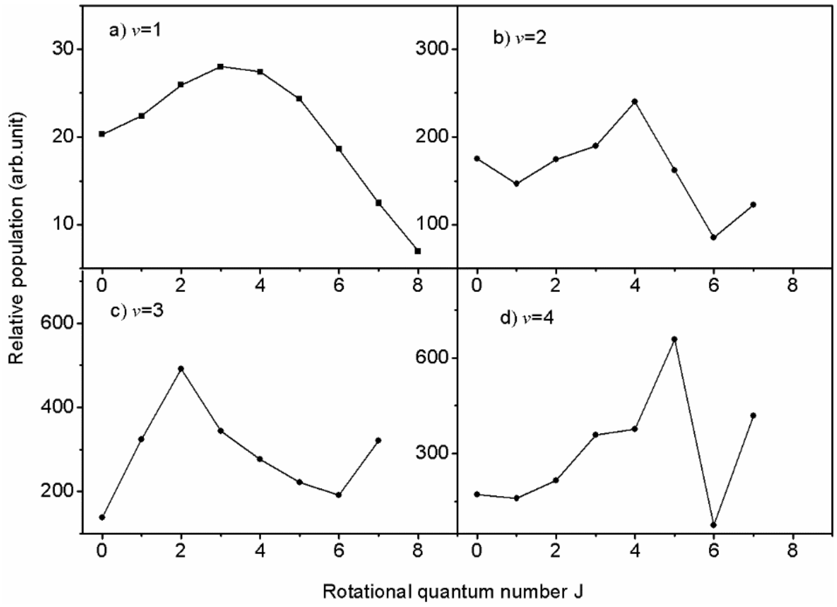

Supplementary Figure 2. Rotational state distributions of H_2 products from H_2O photolysis. Rotational state population distributions for $H_2(X)$ products with the vibrational level *v*=1 (a), *v*=2 (b), *v*=3 (c), and *v*=4 (d) in the $O(^1S) + H_2$ channel at 107.65 nm photolysis.

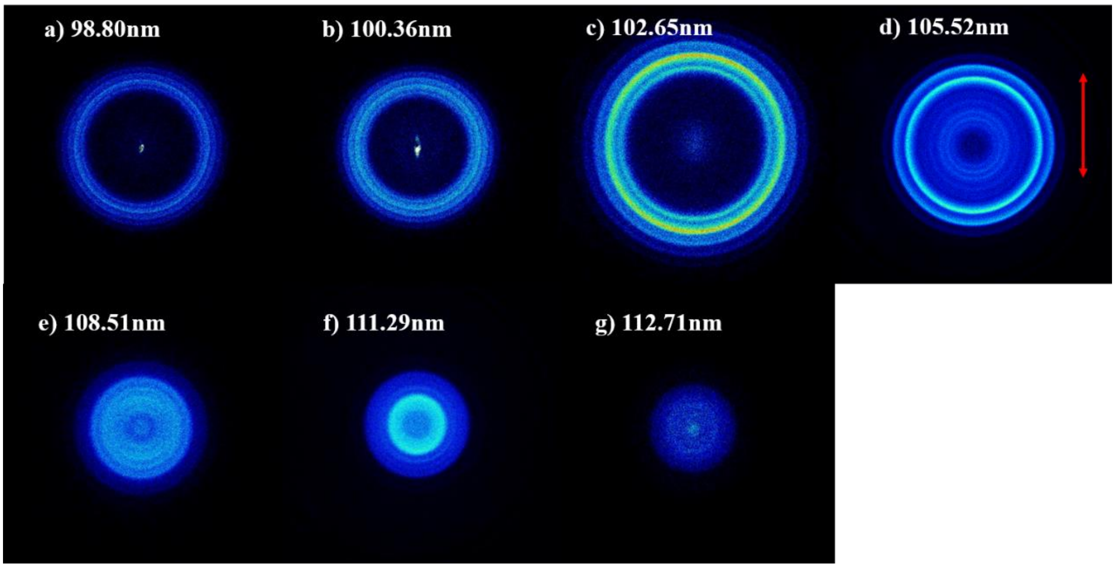

Supplementary Figure 3. Wavelength dependent time-sliced velocity map images from D_2O photolysis. Time-sliced images of the $O(^1S)$ photoproducts from photodissociation of D_2O at (a) 98.80, (b) 100.36, (c) 102.65, (d) 105.52, (e) 108.51, (f) 111.29, and (g) 112.71 nm. The red double arrow indicates the polarization direction of the dissociation laser. The ring features correspond to the rovibrational states of the coincident $D_2(X, v)$ product. It is noted that the total voltage applied on the ion optics is 1000 V for the photolysis wavelengths 98.80 nm and 100.36 nm, and 500 V for the other wavelengths.

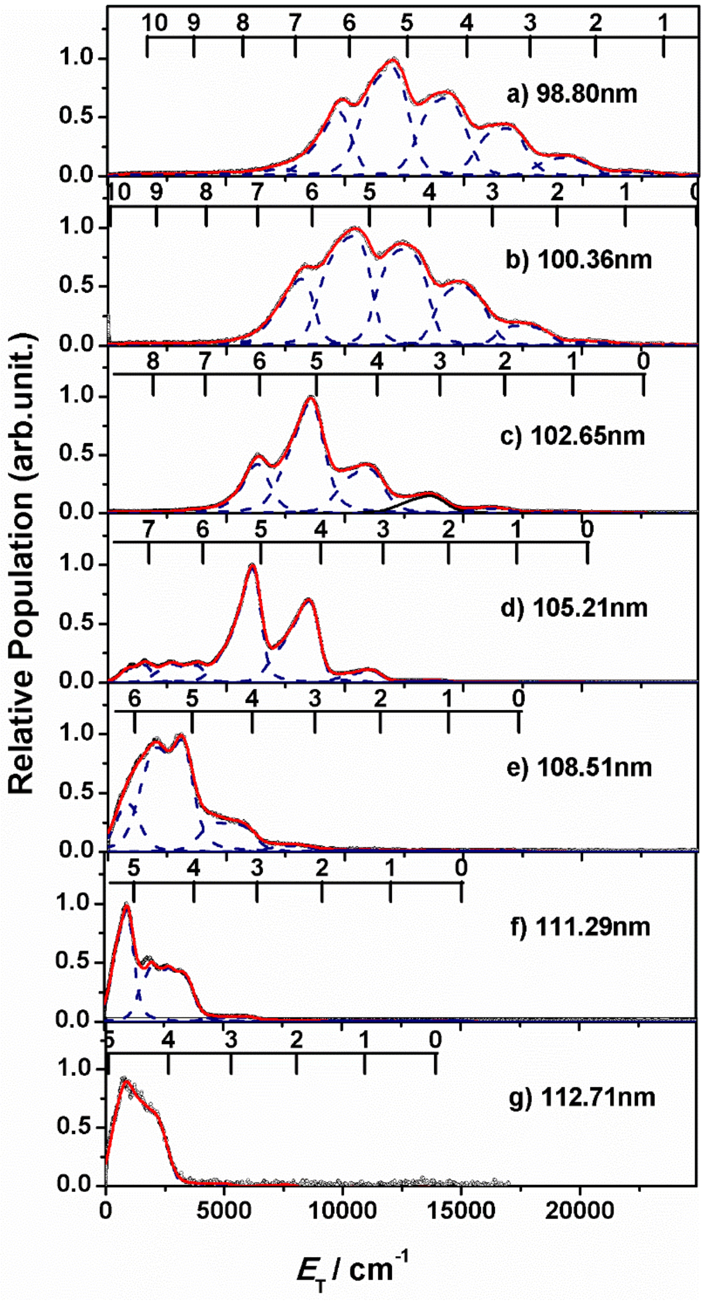

Supplementary Figure 4. Wavelength dependent product kinetic energy distributions from D_2O photolysis. The product total kinetic energy distribution (E_T) spectra derived from the images shown in Supplementary Figure 3, in red, along with the best-fit simulation of the spectra, in navy. The superposed combs indicate the E_T values associated with formation of the various vibrational states of D_2(X).

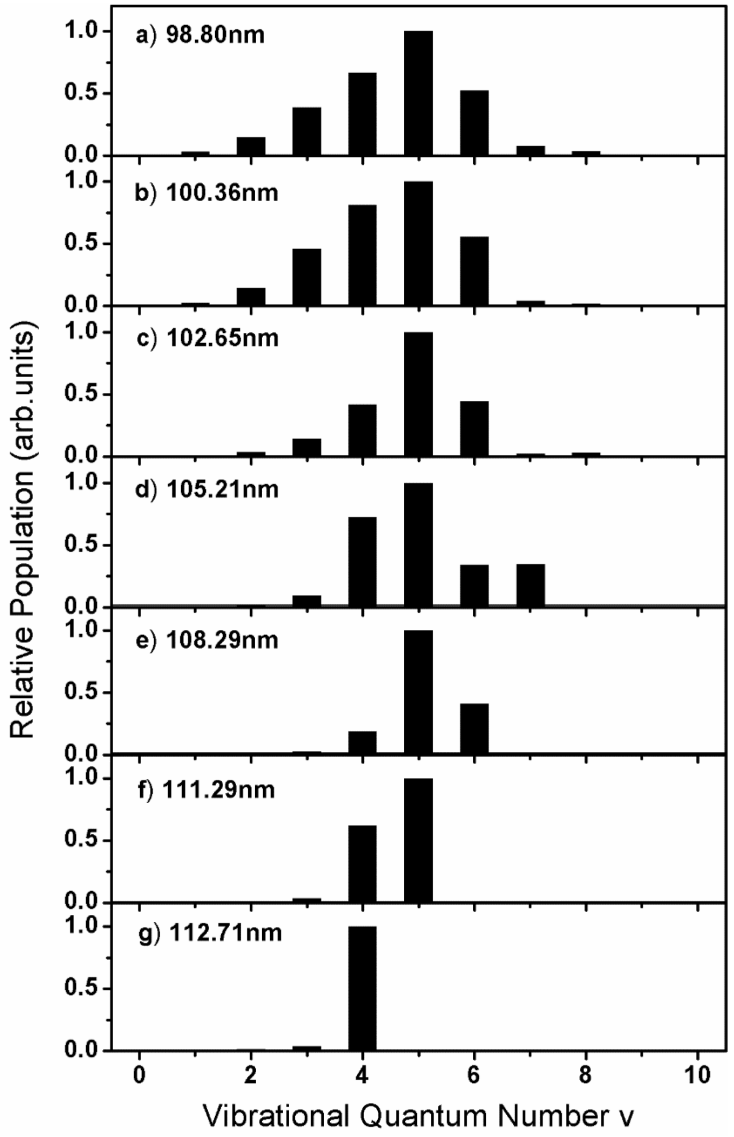

Supplementary Figure 5. Vibrational state distributions of D_2 from D_2O photolysis. Relative populations of different vibrational states of the $D_2(X)$ products in the $O(^1S)$ + D_2 channel from the $O(^1S)$ detection at seven photolysis wavelengths.

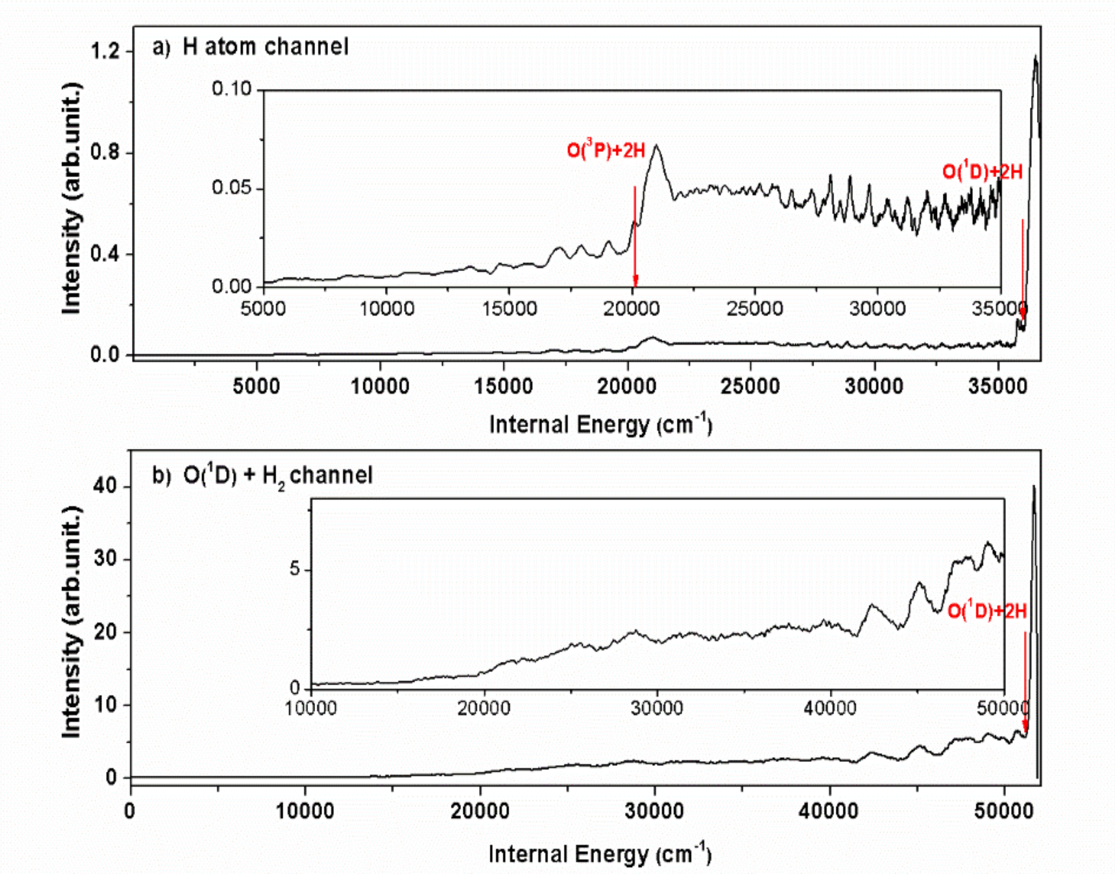

Supplementary Figure 6. Photodissociation branching ratio measurements from H_2O photodissociation at 107.5 nm. a) The product internal energy distribution from H_2O photodissociation at 107.5 nm with the detection axis at 54.7° (magic angle) to the VUV FEL laser polarization direction. The spectrum is converted from the time-of-flight (TOF) spectrum recorded by the H atom Rydberg tagging TOF technique. Four dissociation channels are shown in this feature: H + OH(X), H + OH(A), $O(^3P)$ + 2H, and $O(^1D)$ + 2H. The threshold energies for the three-body channels $O(^3P)$ + 2H and $O(^1D)$ + 2H have been marked by red arrows. b) The product internal energy distribution from H_2O photodissociation at 107.5 nm converted from $O(^1D)$ fragment images, which was recorded by using the VUV FEL - TSVMI setup. Two dissociation channels are shown in this feature: $O(^1D) + H_2$ and $O(^1D)$ + 2H. The threshold energy for the $O(^1D)$ + 2H channel has also been marked by the red arrow.

Supplementary Note 1

The available energy deposited in vibration of H_2 molecules can be deduced from the vibrational state population distributions of H_2 by using the equation,

$<f_v> = \sum T_v \times P(v)$,   (S1)

Where f_v is the average vibrational energy, T_v is the term value of the vibrational level v of H_2, and $P(v)$ is the relative population of H_2 in the vibrational state v.

Similarly, the available energy deposited in translation can be deduced from the total kinetic energy spectra by using the equation,

$<f_T> = \sum E_T \times P(E_T)$,   (S2)

Where f_T is the average translational energy, E_T is the total kinetic energy distribution, and $P(E_T)$ is the relative population as a function of the translational energy.

While the average rotational energy can be obtained by using the equation,

$<f_r> = 1 - <f_T> - <f_v>$,   (S3)

Supplementary Note 2

Electronic Structure calculations. The *ab initio* calculations of the four electronic states ($\tilde{X}$, $\tilde{B}$, $\tilde{D}$, and $\tilde{E}'$) of H_2O are performed at the internally contracted multi-reference configuration interaction corrected with Davidson correction (ic-MRCI+Q)[2-4] level using state-averaged completed active space self-consistent field (SA-CASSCF)[5,6] wave function with active space (6*e*, 13*o*) as reference by the MOLPRO package.[7] The basis set used for oxygen is correlation-consistent polarized valence quadruple-zeta with explicitly correlated method level[8] supplemented with spd diffuse functions. For hydrogen, the basis set is correlation-consistent polarized valence quadruple-zeta[9] supplemented with *sp* diffuse functions.

Potential energy surfaces. The non uniform grid in the H_2O internal coordinate (θ_{HOH}, R_{OH1}, and R_{OH2}) is selected to construct the PESs. The θ_{HOH} ranged from 0 ° to 180.0 °, and the OH bond lengths ranged from 0.6 Å to 3.0 Å with R_{OH1} smaller than R_{OH2}. The Gaussian Process Regression method[10-12] were used for fitting the PESs with permutation invariant polynomials[13-16] (PIPs). In total, about 900 *ab initio* points were well selected to construct the PESs of these four electronic states. The RMSEs for the final PESs of the four states were smaller than 10 meV.

Supplementary Note 3

The quantum yield estimation of the $O(^1S) + H_2$ channel. As mentioned in the main text, six fragmentation processes (1) – (6) following VUV photoexcitation of H_2O should be thermodynamically accessible. Recently, we have reported the photofragment translational spectroscopy (PTS) measurements of the H atoms from H_2O photolysis in the wavelength range of $90 \leq \lambda \leq 110$ nm.[17] Supplementary Fig. S6(a) shows such internal energy spectrum converted from the PTS spectrum at 107.5 nm with the detection angle of 54.7° to the VUV FEL laser polarization direction. This feature contains all of H atom elimination channels (1) – (4). The sharp structures have been assigned to ro-vibrational levels of the OH product in the A and X states. In addition to these sharp structures, two broad features in the distributions have ascribed to three body dissociation channels (3) and (4). Best simulation of Fig. S6(a) returns to branching ratios of H + OH(X), H + OH(A), $O(^3P)$ + H + H, and $O(^1D)$ + H + H.

On the other hand, we have applied the VUV FEL-TSVMI apparatus to detect the channel (5). The VUV FEL output was used to excited H_2O molecules to a dissociative electronic state, while the subsequent $O(^1D)$ fragments were then resonantly ionized at 92.2 nm, which was generated by a table-top VUV source from the four-wave sum frequency scheme.[18] The internal energy spectrum converted from the $O(^1D)$ ion images recorded at 107.5 nm is shown in Fig. S6(b). This feature involves two dissociation channels (4) and (5). The structures can be assigned to the vibrational states of H_2 product, while the highest peak located above the H_2 dissociation limit (marked by the red arrow) is ascribed to three body dissociation channel (4). Best simulation of Fig.

S6(b) returns to branching ratios of O(^{1}D) + H_2 and O(^{1}D) + H + H. Due to the same component of the channel (4) in Fig S6(a) and (b), the branching ratios of channels (1) – (5) can be deduced. These experimental details will be presented in the proceeding paper.

The next step is to obtain the branching ratio of channels (5) and (6). Since the O(^{1}S) atom was detected by 121.7 nm photon which was generated by four wave difference frequency scheme, while the O(^{1}D) atom was detected by 92.2 nm photon obtained by four wave sum frequency scheme, the detection efficiencies of the O(^{1}S)/O(^{1}D) atoms are not immediately clear. According to previously reported by Ng et al.[19], the VUV difference generation is about 10 times more efficient than the VUV sum-frequency generation. Based on the theoretical calculations[20], the transition probabilities for the above excitation processes involving O(^{1}S) and O(^{1}D) were reported to be 2.06×10^8 s^{-1} and 1.23×10^8 s^{-1}. Convoluting the VUV generation efficiency and the transition probabilities, the detection efficiency for the O(^{1}S) and O(^{1}D) can be estimated to be 20.6:1.23=16.75:1.

It is cautioned that the Doppler effect in the ion detection and the space overlap between the pump and probe light will affect the detection efficiency, and will cause large uncertainty. Finally, the branching ratio between the O(^{1}S) + H_2 and O(^{1}D) + H_2 channels can be obtained by measuring them under the same experimental conditions. Considering the detection efficiency, the measurements return the branching ratio of channels (6) and (5) to be ~1: 3.4. We have already known the branching ratio of channels (1) – (5) from the supplementary Fig.6, thus the quantum yield of the O(^{1}S) +

H_2 channel can be estimated, ~16±8% at 107.5 nm. The large uncertainty comes from the calibration of the detection efficiency of the $O(^1S)/O(^1D)$ atoms. At the wavelengths longer than 107.5 nm, however, the channel (4) is not open (D_{th}=11.48 eV). It is hard to compare the signal intensity of the H atom channel and the $O(^1D) + H_2$ channel.

Supplementary References:

1. Lee, L. C. & Suto, M. Quantitative photoabsorption and fluorescence study of H_2O and D_2O at 50-190nm. *Chem. Phys.* **110**, 161-169 (1986).
2. Shamasundar, K. R., Knizia, G. & Werner, H. J. A new internally contracted multi-reference configuration interaction method. *J. Chem. Phys.* **135**, 054101 (2011).
3. Knowles, P. J. & Werner, H.-J. An efficient method for the evaluation of coupling coefficients in configuration interaction calculations. *Chem. Phys. Lett.* **145**, 514-522 (1988).
4. Davidson, E. R. & Silver, D. W. Size consistency in the dilute helium gas electronic structure. *Chem. Phys. Lett.* **52**, 403-406 (1977).
5. Werner, H. J. & Knowles, P. J. A second order multiconfiguration SCF procedure with optimum convergence. *J. Chem. Phys.* **82**, 5053-5063 (1985).
6. Knowles, P. J. & Werner, H.-J. An efficient second-order MC SCF method for long configuration expansions. *Chem. Phys. Lett.* **115**, 259-267 (1985).
7. Werner, H. J., Knowles, P. J., Lindh, R., Manby, F. R. & Schütz, M. *MOLPRO , version 2006.1, a package of ab initio programs*.
8. Peterson, K. A., Adler, T. B. & Werner, H.-J. Systematically convergent basis sets for explicitly correlated wavefunctions: The atoms H, He, B–Ne, and Al–Ar. *J. Chem. Phys.* **128**, 084102 (2008).
9. Dunning, T. H. Gaussian basis sets for use in correlated molecular calculations. I. The atoms boron through neon and hydrogen. *J. Chem. Phys.* **90**, 1007-1023 (1989).
10. Cui, J. & Krems, R. V. Gaussian process model for collision dynamics of complex molecules. *Phys. Rev. Lett.* **115**, 073202 (2015).

11. Kolb, B., Marshall, P., Zhao, B., Jiang, B. & Guo, H. Representing global reactive potential energy surfaces using gaussian processes. *J. Phys. Chem. A* **121**, 2552-2557 (2017).

12. Yuan, D. et al. Observation of the geometric phase effect in the H + HD → H_2 + D reaction. *Science* **362**, 1289-1293 (2018).

13. Braams, B. J. & Bowman, J. M. Permutationally invariant potential energy surfaces in high dimensionality. *Int. Rev. Phys. Chem.* **28**, 577-606 (2009).

14. Jiang, B., Li, J. & Guo, H. Potential energy surfaces from high fidelity fitting ofab initiopoints: the permutation invariant polynomial - neural network approach. *Int. Rev. Phys. Chem.* **35**, 479-506 (2016).

15. Jiang, B. & Guo, H. Permutation invariant polynomial neural network approach to fitting potential energy surfaces. *J. Chem. Phys.* **139**, 204103 (2013).

16. Bartók, A. P. & Csányi, G. Gaussian approximation potentials: a brief tutorial introduction. *Int. J. Quantum Chem.* **115**, 1051-1057 (2015).

17. Chang, Y. et al. Three body photodissociation of the water molecule and its implications for prebiotic oxygen production. *Nat. Commun.* **12**, 2476 (2021).

18. Song, Y. et al. Photodissociation of CO_2 between 13.540 eV and 13.678 eV. *Phys. Chem. Chem. Phys.* **16**, 563-569 (2014).

19. Lu, Z., Chang, Y. C., Yin, Q. Z., Ng, C. Y. & Jackson, W. M. Evidence for direct molecular oxygen production in CO_2 photodissociation. *Science* **346**, 61-64 (2014).

20. Butler, K. & Zeippen, C. J. Oscillator strengths for allowed transitions in neutral oxygen : an assessment of the opacity project data accuracy. *J. Phys. IV* **01**, C1-141-C141-152 (1991).
